# Phenotype and psychometric characterization of Phelan-McDermid syndrome patients: pioneering towards personalized medicine

**DOI:** 10.3389/fpsyt.2025.1511962

**Published:** 2025-03-04

**Authors:** Julián Nevado, Filippo Ciceri, Cristina Bel-Fenellós, Jair A. Tenorio-Castaño, Tamara Maes, Jordi Xaus, Carlos Buesa, Pablo Lapunzina

**Affiliations:** ^1^ Instituto de Genética Médica y Molecular (INGEMM)-Instituto de Investigación del Hospital Universitario La Paz (IdiPaz), Hospital Universitario La Paz, Madrid, Spain; ^2^ Centro de Investigación Biomédica en RED de Enfermedades Raras (CIBERER), Madrid, Spain; ^3^ ITHACA, European Reference Network on Rare Congenital Malformations and Rare Intellectual Disability, Hospital La Paz, Madrid, Spain; ^4^ Oryzon Genomics SA. Cornellà de Llobregat, Barcelona, Spain; ^5^ Dpto. Investigación y Psicología en Educación, Facultad de Educación, Universidad Complutense, Madrid, Spain

**Keywords:** Phelan McDermid, personalized medicine, aggressively, cognitive-behavioral aspects, genomic medicine, *SHANK3* gene

## Abstract

**Introduction:**

Phelan-McDermid syndrome (PMS) is a genetic disorder caused by the loss of the terminal region of chromosome 22 or by pathogenic or likely-pathogenic variants in *SHANK3* gene. Individuals with PMS are affected by a variable degree of intellectual disability, delay or absence of speech, low muscle tone, motor delay epilepsy, and autistic features. We have performed an observational trial aimed to psychometrically characterize individuals carrying deletions or pathogenic variants in *SHANK3*, to eventually build a foundation for a subsequent precision psychiatry clinical trial with vafidemstat, a LSD1 inhibitor in Phase II clinical development.

**Methods:**

We have conducted a pilot study to clinically characterize the profile of 30 subjects, all diagnosed of molecularly confirmed PMS. Subjects were phenotypically characterized by applying different psychometric scales, including Repetitive Behavior Questionnaire (RBQ), Vineland Adaptive Behavior Scales, ADOS-2, the Battelle developmental inventory screening test and the Behavior Problems Inventory (BPI). Nineteen patients were included in the pilot study, followed by additional 11 individuals in the validation set.

**Results:**

Unsupervised hierarchical clustering of the collected psychometric data identifies three groups of patients, with different cognitive and behavioral profile scores. Statistically significant differences in deletion sizes were detected comparing the three clusters (corrected by gender), and the size of the deletion appears to be positively correlated with ADOS and negatively correlated with Vineland-A and -C scores. No correlation was detected between deletion size and the BPI and RBQ scores.

**Discussion:**

This analysis presents new data on the best potential endpoints, for a future clinical study exploring vafidemstat actionability for *SHANK3*-associated psychiatric disorders, constituting a good example of how Precision Medicine may open new avenues to understand and treat Central Nervous System (CNS) disorders, pioneering individual management in PMS.

## 1 Introduction

Phelan-McDermid syndrome (PMS, OMIM#606232), formerly known as 22q13.3 syndrome or 22q13 deletion, is an infrequent neurogenetic disorder that affects the telomeric-end of the long arm of chromosome 22. At the molecular level, this syndrome is characterized by a partial or complete loss of genetic material within the 22q13.3 region, which includes the *SHANK3* gene (1, 2). Pathogenic and likely pathogenic variants affecting this gene may explain many of the clinical features presented in PMS. In fact, *SHANK3* seems the main gene involved in PMS, and its dysfunction is associated with many of the clinical symptoms observed in the patients, suggesting that this gene is a major player with a significant role in neurological and synaptic development in humans (3, 4). The connection between underlying genetics and clinical phenotype in PMS remains a complex puzzle that researchers seek to unravel, in order to improve the understanding and management of this condition, and to gain knowledge for potential therapies.

At clinical level, PMS is associated with a variety of clinical manifestations that directly impact the neurological and physical development of those individuals suffering from this syndrome. In fact, one of the most distinctive features of PMS is the moderate to severe intellectual disability, which is present in more than 95% of the cases (2, 5, 6). In addition, affected people usually have delays in the development of speech, fine and gross motor skills, as well as problems in motor coordination. In addition, other common physical features are observed, such as distinctive faces, gastrointestinal problems, muscle hypotonia, limb abnormalities, seizures and sleep anomalies (7, 8).

Patients with PMS can also manifest with autism spectrum disorders (ASD) (9–13). In particular, the relationship between loss of the 22q13.3 region and autism symptoms represents a significant area of interest for medical research. The presence of ASD has also attracted significant interest, underscoring the clinical complexity and need for a multidisciplinary approach in the care of affected individuals. PMS is also associated with a variety of behavioral features, which can vary considerably among patients, due to the highly heterogeneous clinical expression. Worth of mention are: communication difficulties, socialization problems, sensory sensitivity, anxiety responses, stereotyped behaviors, difficulties in adapting to change as well as aggression, self-harm, and difficulties managing emotions (14–18). It is important to note that the behavioral profile can vary widely among people with PMS. Each individual is unique and may present different combinations of behavioral characteristics. Early intervention and support, as well as an individualized approach, are essential to address behavioral challenges and improve the quality of life of these individuals.

Currently, there are no pharmacological treatments approved for PMS and the therapeutic approach mainly focuses on treating specific symptoms via speech, occupational and physical therapies, as well as educational interventions focused on specific individual needs. As research progresses, it is expected to open new avenues for more targeted and effective personalized interventions, for those living with this rare genetic condition (see 6 for a review of past, ongoing and future clinical trials in PMS). Functional *in vitro* assays showed that *SHANK3* disruption in mice leads to dysfunction of synaptic transmission, which can be restored by epigenetic regulation with both lysine-specific demethylase 1 (LSD1) and/or Histone deacetylases (HDAC) inhibitors (19–22). In fact, epigenetic dysregulation has been proposed to be an important mechanism in the pathogenesis of schizophrenia and autism (21, 22). Vafidemstat is an LSD1 inhibitor in Phase II clinical development that has shown to be effective in restoring memory and cognition defects or reducing agitation and aggression in several animal models in pre-clinical studies (23). Moreover, this investigational drug has exhibited a very good safety profile in Phase I and Phase II clinical trials and promising efficacy insights in CNS-related indications, such as schizophrenia, Alzheimer´s disease or disturbances of personality disorders (24–28). Thus, vafidemstat holds exciting therapeutic potential to be used in individuals with PMS. In summary, this study can inform on the best endpoints and provides insights for patient stratification strategies for a future clinical study to explore vafidemstat actionability for *SHANK3*-associated psychiatric disorders.

## 2 Materials and methods

### 2.1 The study

This study has to be considered a proof of concept. It was designed only for informative purposes and no formal hypothesis or study size calculation was applied. Indeed, the proposed proof of concept study is not contingent solely on the clustering findings but is part of a broader effort to explore personalized therapeutic approaches for PMS.

### 2.2 Cohort

All individuals selected for this study (n = 30) have been previously reported as having PMS by our group and they all belong to the Spanish Population (5). The female/male ratio is 1:1, with ages ranging from 5.2 to 40.25 years-old. Descriptive statistics for demographic data are shown in **Table 1**.

**Table 1:** Demographic data for the whole cohort (n: 30 individuals).

Demographic data			Test set	Validation set
n° of patients	30		19	11
Sex	Male	15 (50%)	11 (58%)	4 (36.4%)
	Female	15 (50%)	8 (42%)	7 (63.6%)
Age	Median	18.75	19.16	10.33
	(Min/Max)	(5.2, 40.25)	(12.1, 40.2)	(5.2, 40.25)
Type mutation	Deletion	29 (96.7%)	18 (94.7%)	11 (100%)
	Point Mut	1 (3.3%)	1 (5.3%)	
Size of deletion (Mb)	Median	2.04	2.19	2.04
	(Min/Max)	(0.023, 9.56)	(0.023, 9.56)	(0.094, 8.82)
Localization of deletion/mutation	Interstitial	1 (3.3%)	1 (5.3%)	0
	Origin translocation	1 (3.3%)	0	1 (9.1%)
	Frameshift	1 (3.3%)	1 (5.3%)	0
	Terminal	21 (70%)	14 (73.7%)	7 (63.6%)
	Terminal Mosaic	6 (20%)	3 (15.8%)	3 (27.3%)
Medication regimen at assessment visit	Not known	4 (13.3%)	3 (15.8%)	1 (9.1%)
	No medication	7 (23.3%)	5 (26.3%)	2 (18.2%)
	One drug	6 (20%)	2 (10.5%)	4 (27.3%)
	Drug Combo	13 (43.3%)	9 (47.4%)	4 (27.3%)

The cohort was split in two groups: a training set used for the initial unsupervised hierarchical clustering (19 individuals) and a validation set (11 individuals) to confirm the observations from the first set of patients. Most of the individuals have gene deletions, but one patient carries a pathogenic *SHANK3* sequence variant (frameshift variant).

Regarding our range of age in this study, although our Spanish cohort (210 individuals) is predominantly a child cohort (around 70%), we have tried to select individuals predominantly adults >16 years old of age in order to be able to test the drug (vafidemstat) in a future clinical trial. This drug is not currently approved in children. In fact, the training part of this proof of concept with 19 individuals the average age is almost 21 years, and in the validation part (included any child to rule out the age effect), with an average of almost 15 years old, close to the target age of 16 years. In both cases, the effect of age and current pharmacological treatments does not seem to affect the generation of the clusters obtained.

### 2.3 Neuropsychological assessment instruments

Subjects were phenotypically characterized applying different psychometric scales, including Repetitive Behavior Questionnaire (RBQ; 29), Vineland Adaptive Behavior Scales-3 (30), Autism Diagnostic Observation Schedule–2 (ADOS-2; 31), the Battelle developmental inventory screening test (32) and the Behavior Problems Inventory (BPI; 33). Trained psychologists conducted the assessments, mainly at the individual’s home. The hospital ethics committee approved the study (PI-474, June 2020), and caregivers gave informed consent. The selection of the measurement instruments mentioned above has been carried out taking into account the characteristics of the participants and the evaluation procedure itself, which involves at least three procedures: structured examination, observation, and informational interview. By collecting data through this process, the information is contrasted and adjusted to the actual level of the individuals, pointing out the difficulties derived from the evaluation of subjects with intellectual disabilities.

It has to mention that IQ assessments were not conducted in this study due to their limited validity in individuals with moderate-to-severe intellectual disability, which is characteristic of PMS. Instead, we utilized psychometric tools “validated” (14) for this population, including the Vineland Adaptive Behavior Scales, Behavior Problems Inventory, and Autism Diagnostic Observation Schedule (ADOS-2). These measures capture behavioral and adaptive functioning more reliably in individuals with severe cognitive impairments. In addition, we have previously documented experience with BPI, and RBQ tests in two other syndromes, such as Cri du Chat (34), and Wolf-Hirschhorn syndrome (in preparation).

### 2.4 Statistical analysis

Unsupervised hierarchical clustering (35) is one of the most common strategies to identify patterns in data pools, without any prior knowledge about identifying characteristics, properties or classifications (unlabeled data). This algorithm compares each element of the dataset and calculate a matrix of distances to build a hierarchical structure, grouping together objects with similar features in common clusters. A dendrogram is a representation of this hierarchical organization, allowing to visualize each cluster as node in a nested tree structure. Dendograms and hierarchical clustering have been successfully used to clustered patients according to different gene expression profiles (36) or different clinical features (37).

The structure of a complex dataset can be also investigated by reduction dimensionality techniques such as Principal Component Analysis (PCA; 38). In essence, PCA is an orthogonal linear transformation (rotation) of the initial dimensions of the dataset to identify a new set of coordinates (called principal components), ordered according to the percentage of variability explained. By selecting the first two or three principal components, multidimensional datasets can be represented as 2- or 3-D scatterplots. Gene expression analysis frequently involve PCA to visualize differences between two sets of samples characterized by different gene expression profiles (39).

The ANOVA and PCA analyses were performed using the ‘anova’ and ‘prcomp’ functions from R (version 4.2.2). Before PCA, data were centered and scaled. Hierarchical clustering and heatmap visualization were obtained with the ‘heatmap’ function from the ‘ComplexHeatmap’ R package, after scaling the results by columns, to facilitate comparison between different scoring systems. The package ‘rpart’ was applied for tree-based classification with minsplit set to 2. Base R and ‘ggplot2’ were used for graphical visualization and for calculating regression lines and Pearson correlation (with associated p-values).

While this sample size does not meet conventional thresholds for high-powered studies, it aligns with norms in rare disease research. Statistical methods like hierarchical clustering and ANOVA were chosen to maximize the interpretability of the data within these constraints. Future studies will require larger cohorts to validate the findings further.

### 2.5 Limitations

Given the rarity of PMS, the sample size (n=30) is comparable to those in other studies of ultra-rare diseases. Statistical power for the primary clustering analysis was estimated by *post hoc* analysis and significant differences across clusters were observed in behavioral and cognitive scales. However, the small sample size limits the inclusion of additional covariates without risking statistical overfitting, necessitating larger follow-up studies.

## 3 Results

### 3.1 Unsupervised hierarchical clustering

Cognitive and behavioral evaluation was performed initially on 19 PMS patients (mean age 21 years, min-max: 12-40, **Table 1**), according to the Battelle, Behaviors Problem Inventory (BPI-FR and BPI-GR), Repetitive Behaviors Questionnaire (RBQ), Vineland E/C and ADOS Sum psychometric scales. For each scale, we considered both total scores and sub-scores for specific components.

**Figure 1:**
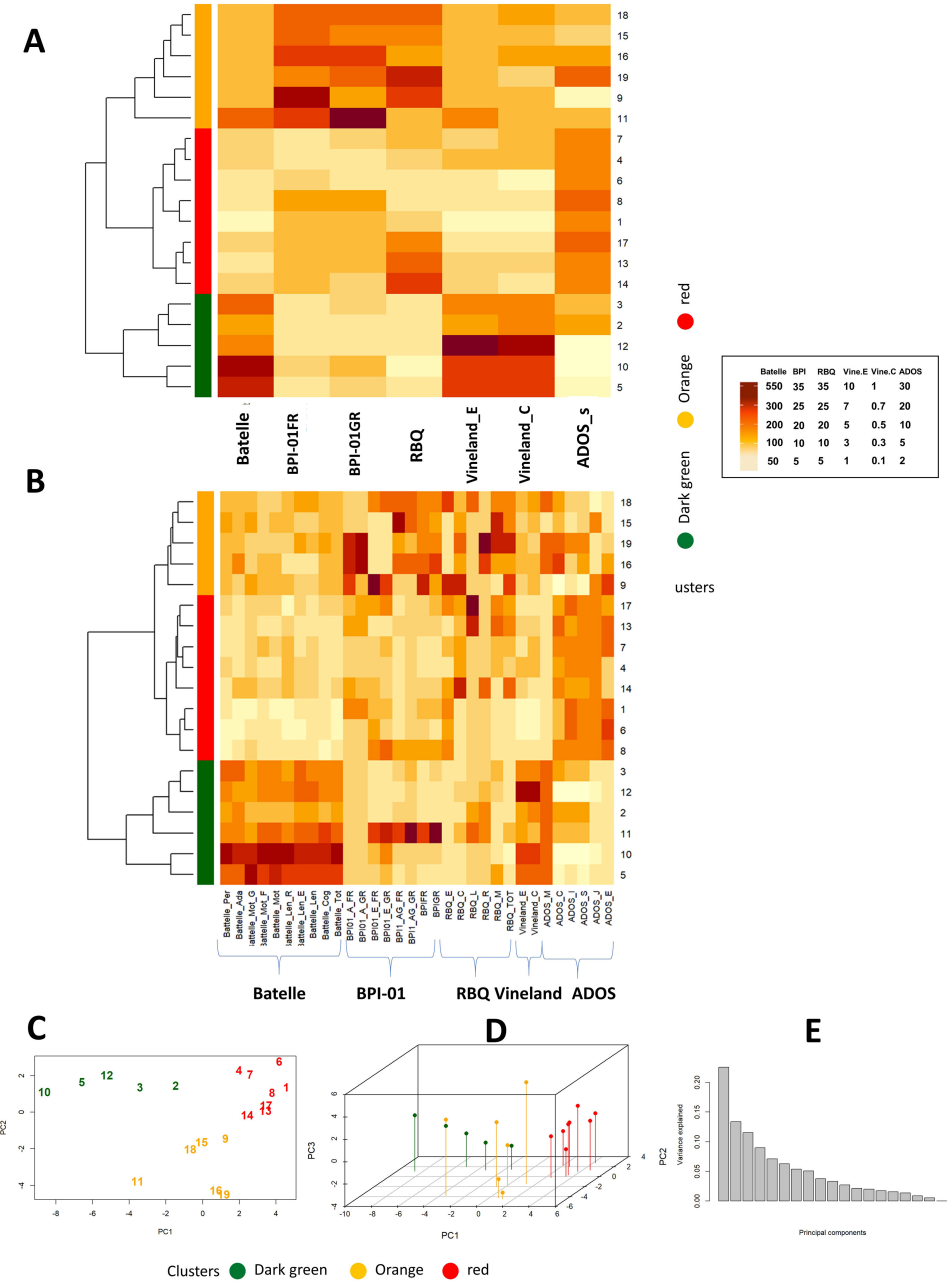
**(A, B)** Heatmaps for total scores for psychometric scales alone **(A)** or complemented with specific sub-scores **(B)**. Vertical bars for the cluster and similarity dendograms are reported on the left side, patient IDs on the right side. C, D) PCA analysis considering the first 2 **(C)** or 3 **(D)** main components. Patients IDs and cluster of origin are reported in the figure. **(E)** Percentage of variance explained by each individual Principal Component. Dark brown means higher numbers (arbitrary units) in the different psychometric scales (see the specific scale), and light browns means lower numbers (arbitrary units) in the scales. Batelle_Per, personal; Batelle_Adap, adaptative; Batelle_G, gross motility; Batelle_F, fine motility; Batelle_Mot, total motility; Batelle_Len_R, Receptive language; Batelle_Len_E, Expressive language; Batelle_Len, Total language; Batelle_Con, Total Cognitive area; Batelle_Tot, Total Batelle score; BPI01_A_FR, BPI self-injury frequency; BPI01_A_GR, BPI self-injury severity; BPI01_E_FR, BPI stereotypies frequency; BPI01_E_GR, BPI stereotypies severity; BPI01_AG_FR, BPI aggressive frequency; BPI01_E_GR, BPI aggressive severity; BPIFR, total frequency at BPI (Behavior Problems Inventory) test; BPIGR, total severity at BPI test; RBQ (Repetitive Behavior Questionnaire)_E, Sterotipies score at RBQ; RBQ_C, Compulsive behavior score; RBQ_L, Limited preferences score; RBQ_R, Repetitive speech score; RBQ_M, Monotony score; RBQ_TOT, Total RBQ score;Vineland_E, Vineland test social age, Vineland_C, Vineland test social coefficient; ADOS_M, per module type; ADOS_C, per communication score; ADOS_S, per the sum of the game and social interaction scores; ADOS_J, per playing score; ADOS_E, per stereotype score.

**Figure 2:**
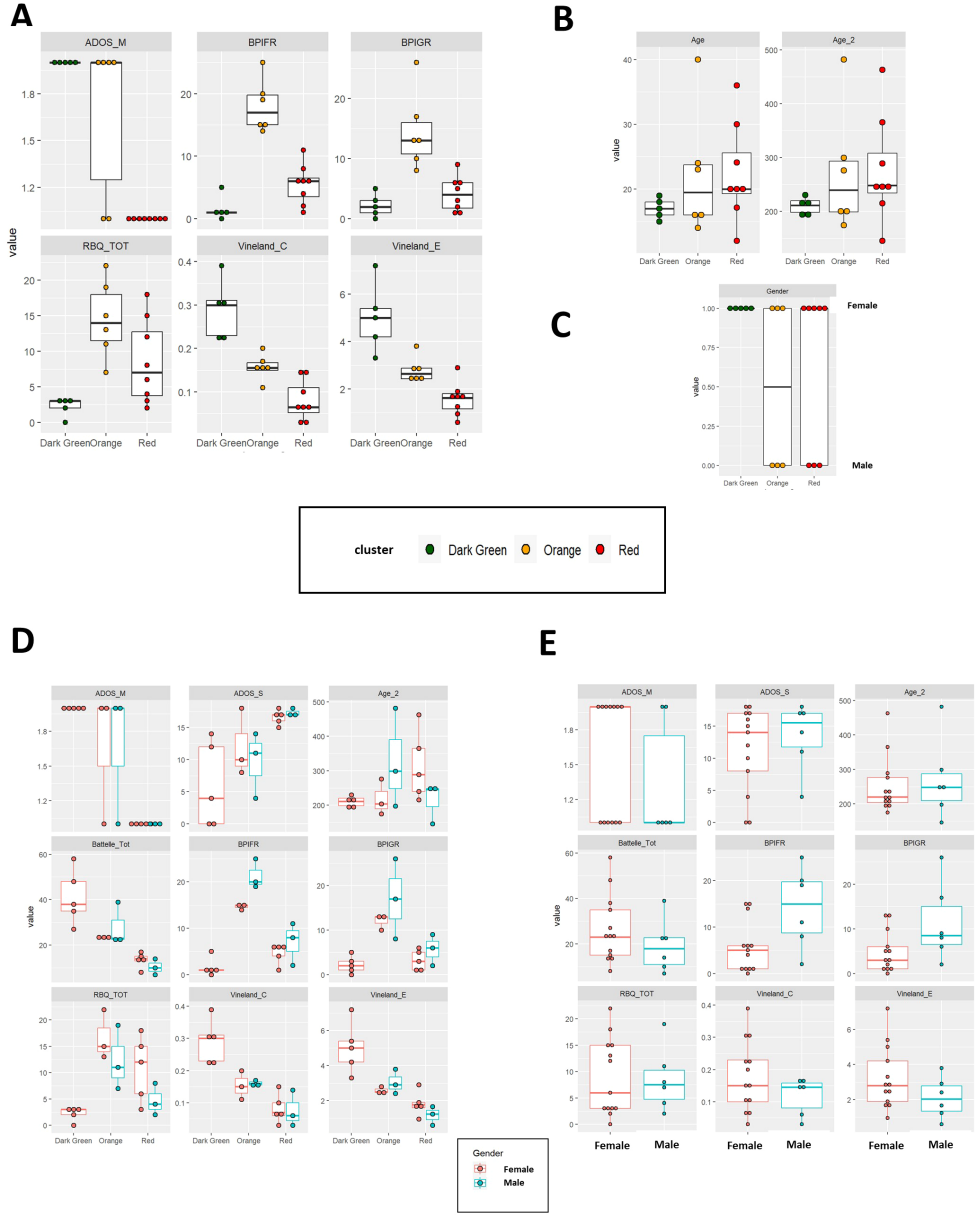
**(A–C)** Comparison of psychometric scales **(A)**, age **(B)** and gender frequency **(C)** between the 3 clusters of patients (color-coded in the figure). **(D)** Boxplot comparing RBQ (Repetitive Behavior Questionnaire) and BPI (Behavior Problems Inventory) scores in male and female patients from the 3 clusters. **(E)** Box plots comparing psychometric scales between the entire set of female (in red) and male patients (in blue).

To identify subgroups of patients characterized by different clinical features, unsupervised hierarchical clustering was performed (see Methods) considering total scores and individual components of each one of the scales described above (respectively **Figures 1A, B**). In both cases, unsupervised hierarchical clustering identified 3 distinct groups of patients, color-coded as “red” (cluster R), “orange” (cluster O) or “green” (cluster G) (**Figure 1**; for more details also see **Supplementary Figure S1**). Remarkably, complementing hierarchical clustering with individual components provided the same subsets of patients obtained considering only total scores (with the exception of patient 11 assigned to cluster “G” or cluster “O” depending on the selected scoring system). This suggests that scoring for specific sub-scales is not providing additional information for clustering and, for this reason, this approach was not considered further. Graphical visualization of the three clusters of patients was performed after retaining either the first two (**Figure 1C**) or three main components (**Figure 1D**) obtained from PCA analysis, explaining respectively up to 36% or 47% of the total variance (**Figure 1E**). When compared, the three clusters of patients display clear differences in term of cognitive scores (**Figure 2A**, **Supplementary Figure S1**) and comparable age distribution (**Figure 2B**).

The “red” cluster is characterized by low Battelle mental age, low Vineland, RBQ and BPI scores. Low RBQ and BPI scores are detected also in the “green” cluster, which in contrast has high Battelle mental age and Vineland scores (i.e. better cognition and mental age). The “orange” cluster resides somewhat in the middle for the Battelle and Vineland scores, while displaying the highest levels of aggression (high BPI) and repeated behaviors (high RBQ). Panels in **Figure 2A** represent between-cluster comparison for each of the scales described above. In line with previous observations, female patients are generally affected by a milder phenotype. None of the male patients were present in the “green” cluster (**Figure 2C**). Also, male patients in the “orange” cluster are characterized by higher levels of aggressive behavior, whereas female patients from the same cluster display a tendency towards to a higher repetitive behavior (**Figure 2D**). Similar conclusions were obtained considering the entire set of patients (**Figure 2E**). The results of 1-way or 2-ways ANOVAs showed that, for all the cognitive scores considered, the differences between the three groups were statistically significant also after adjusting for gender (**Table 2**). Gender-specific differences in terms of BPIFR and RBQ scores were also detected (**Table 2**).

**Table 2:** Results (p-values) for non-adjusted (1-way ANOVA) and gender-adjusted (2-ways ANOVA) comparisons of behavioral and cognitive scales between clusters.

	n.a. cluster p-value	adjusted cluster p-value	adjusted gender p-value
Battelle_Total	0.0000169	0.0000335	0.9304355
BPIFR	0.0000007	0.0000001	0.0115220
BPIGR	0.0002166	0.0001553	0.1151683
RBQ_Total	0.0033619	0.0015591	0.0453811
Vineland_E	0.0000257	0.0000480	0.7614578
Vineland_C	0.0000053	0.0000114	1.0000000
ADOS_M	0.0000394	0.0000743	1.0000000
ADOS_S	0.0014616	0.0021572	0.7988892

BPIFR, total frequency at BPI (Behavior Problems Inventory) test; BPIGR, total severity at BPI test; Vineland_E, Vineland test social age, Vineland_C, Vineland test social coefficient; ADOS-2_M, per module; ADOS_S, per the sum of the game and social interaction scores.

**Figure 3:**
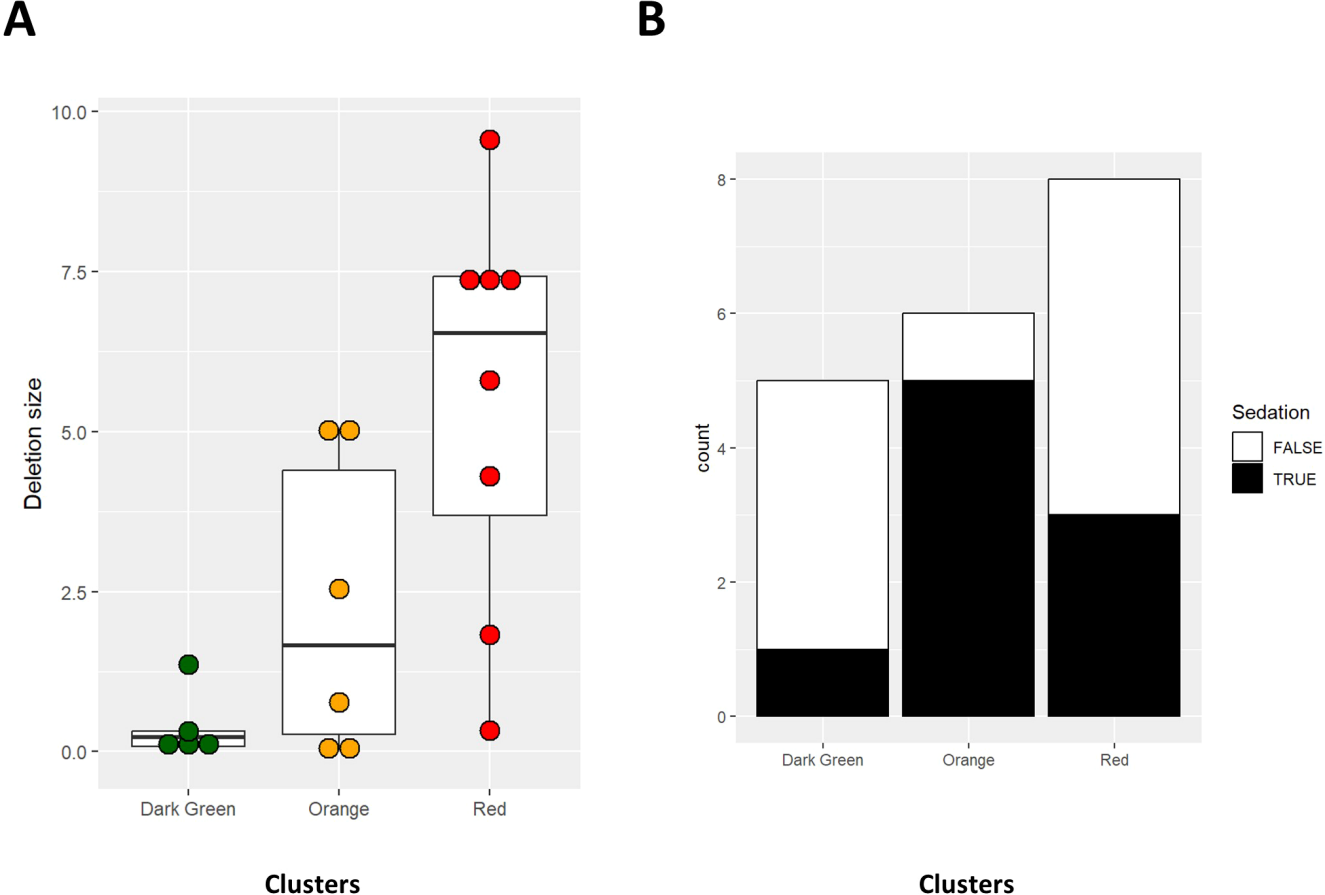
Box plots for deletion size **(A)** and bar charts for administration of sedatives **(B)** in patients from the three clusters.

Deletion size and administration of sedatives were considered as additional factors explaining the differences in behavioral and cognitive scores identified among the three groups. In this small subset of patients, statistically significant differences in deletion sizes were observed comparing the three clusters, also after correcting for gender (**Figure 3A**, **Table 3**). Another possible explanation for the reduced aggression and tendency toward repetitive behavior in severely affected patients may be the higher frequency of sedative treatment in this population (“red”; R cluster). However, administration of sedative treatment is actually higher within the “orange” (O) cluster, which is also characterized by the highest scores for the BPIFR, BPIGR and RBQ total scales (**Figure 3B**). **Table 3** summarizes the results of 1-way and 2-ways ANOVA comparing age and deletion size with and without correction for gender.

**Table 3:** Results (p-values) for non-adjusted (1-way ANOVA) and gender-adjusted (2-ways ANOVA) comparisons of age and deletion size.

	n.a. cluster p-value	adjusted cluster p-value	adjusted gender p-value
Age	0.4124341	0.4353755	0.880625
Deletion size	0.0060842	0.0004335	0.001354

**Figure 4:**
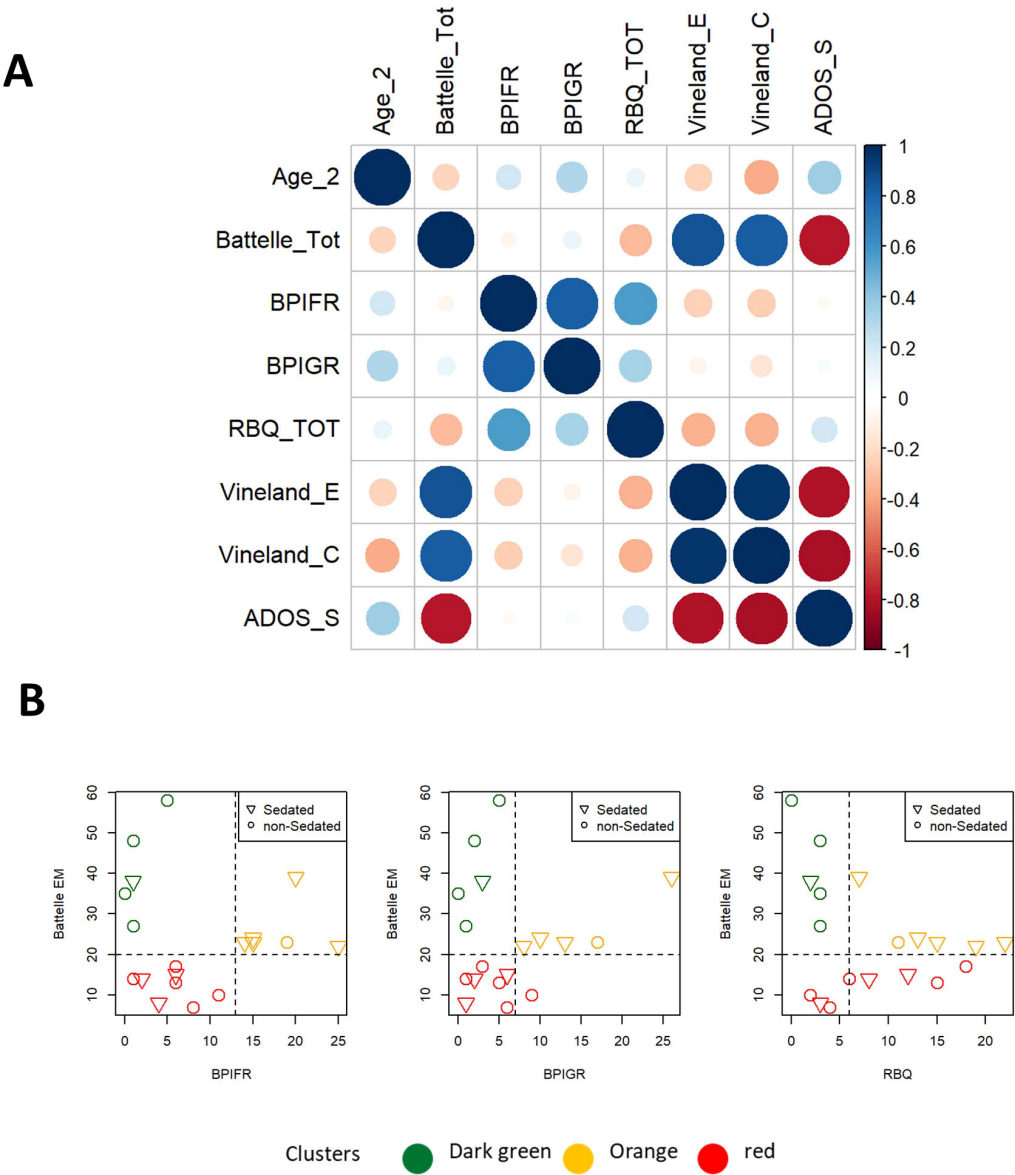
Scatterplot for specific gating strategies defined based on different combinations of Battelle ME (Estimated Motor score at Batelle), RBQ-tot (total Repetitive Behavior Questionnaire) or BPI (Behavior Problems Inventory; BPI-GR for severity or BPI-FR for frequency).

The combined use of psychometric scales such as the Battelle Developmental Inventory, the Repetitive Behavior Questionnaire (RBQ), and the Behavior Problem Inventory (BPI) provides a comprehensive assessment of developmental milestones, repetitive behaviors, and problem behaviors in patients with syndromes or disabilities, enabling a more tailored approach to intervention. Specifically, we focus on mental age, repetitive behavior, and aggression, considering their severity and frequency (assessed by BPI-GR and BPI-FR, respectively). These scales show low correlation among themselves, with the exception of BPI-FR (as shown in **Figure 4A**, **Supplementary Figure S2**). **Figure 4B** assigns patients to the “R,” “O,” and “G” clusters based on three different gating strategies, which compare the Battelle scale (regarding Mental Age) against either RBQ-total or BPI (BPI-GR or BPI-FR). The cut-offs for the “orange” cluster are: Battelle Mental Age > 20, BPI-FR > 13, BPI-GR > 7, and RBQ > 6.

**Figure 5:**
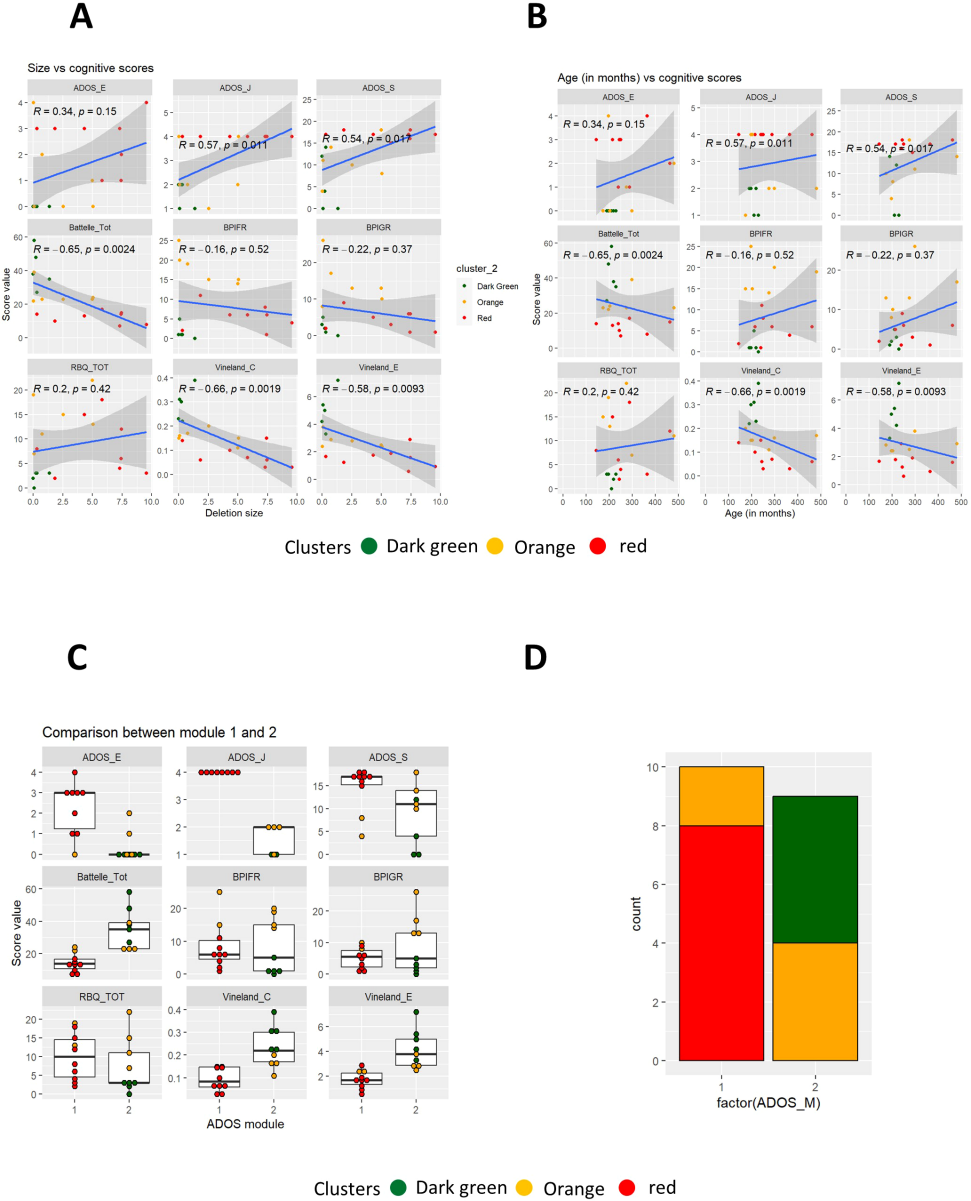
**(A, B)** Correlations plots between psychometric scales and deletion size **(A)** or biological age **(B)**. Cluster are color-coded in figure. Pearson R and p-values are also reported in figure. **(C)** Box plots comparing psychometric scales between patients classified as ADOS-2 module 1 and module 2. **(D)** Frequency of patients from clusters R, O and G among module 1 and module 2 patients.

Deletion size appears to be positively correlated with ADOS and negatively correlated with Batelle, Vineland-E and -C scores. On the other hand, no correlation was detected between deletion size and the BPIFR, BPIGR and RBQ scores (**Figure 5A**). Besides, Battelle, ADOS and Vineland scales displayed correlation with patient age (**Figure 5B**). Patients classified as ADOS -2 module 1 and ADOS-2 module 2 differ in term of ADOS-2, Vineland scores and Battelle scale (Mental Age) (**Figure 5C**). Patients of cluster “G” are classified as module 2, whereas those in cluster “R” as module 1. Patients from cluster “orange” can be found in both subsets (**Figure 5D**).

**Figure 6:**
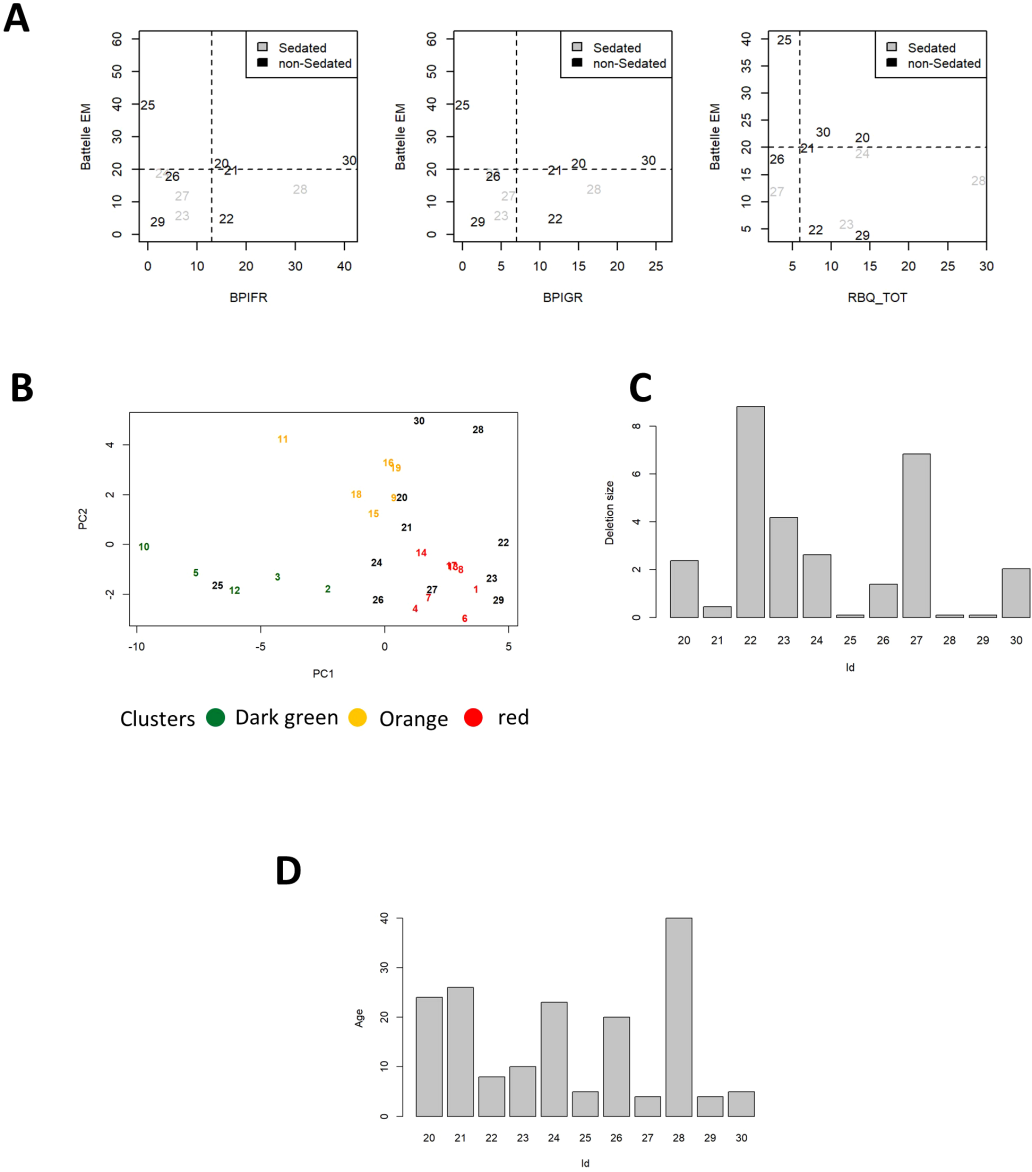
**(A)** Scatterplots for Battelle Mental Age, BPI-GR, BPI-FR and RQB-tot. Cutoffs for classification to the ROG clusters are shown by dashed lines. Patients 1-19 are color coded according to the respective clusters, patients from the first validation set are in black. Dot shape represents administration of sedative treatment (reported in legend). **(B)** Patients 20-30 are plotted according to the values of the first 2 principal components (in black). Patients 1-19 are also plotted in figure color coded according to the cluster of origin. Deletion size **(C)** and age **(D)** of patients 20-30.

The classification based on Battelle scale (Mental Age), BPI and RBQ scales was then validated on an additional set of 11 patients (numerically coded from 20 to 30), not considered for the initial hierarchical clustering analysis (validation set). Patients 20, 21 and 30 are classified as “O” cluster according to all the metrics defined above, whereas patients 22-24 and 26-29 belong to the “R” cluster (because of Battelle; Mental Age, below 20). In the validation set only patient 25 was assigned to the “G” cluster (**Figure 6A**), as he is showing remarkably high Battelle (Mental Age) and low agitation, according to the BPI and RBQ scales respectively. On the other side of the spectrum there is patient 28 (“R” cluster), with very high agitation/aggression and low Battelle (Mental Age). As expected, patient 28 is under sedative medication while patient 25 is not. Patient 26 is borderline between the “R” and “G” cluster, having Battelle age just below 20 (**Figure 6A**). Validation was performed visualizing the distribution of patients on arbitrary axes defined by the first two or three main components (as obtained by PCA). This approach confirmed the proximity of patients 20, 21 and 30 to the “O” cluster (**Figure 6B**), despite having borderline levels of Battelle (Mental Age) and RBQ-tot (only patient 21). For patient 21, this is appreciated only after considering the third principal component in the visualization (data not shown). Patient 25 is in the middle of the “G” cluster, while patients 22, 23, 29 and 27 clearly belong to the “R” one (**Figure 6B**). In line with the previous classifications, patients 24 and 26 fall on the border between the “G” and the “R” clusters. Patient 28 is isolated at an extreme of the plot, highlighting the uniqueness of his/her condition (**Figure 6B**). In line with observations reported for patients 1-19, deletion size is larger than 3 Mb in 3 patients from the “R” cluster (22, 23 and 27) and smaller in patients 24 and 26 (on the edge between the “R” and “G” cluster). The smallest deletion size is reported in patient 25 from the “G” cluster. Interestingly very small deletion sizes are reported also in patients 28 and 29, from the “R” cluster (**Figure 6C**). Mean age for patients 20-30 is 15 years (min-max: 4-40, **Figure 6D**). The disease characteristics of patients from the cluster “O” make them particularly suited for targeted epigenetic therapies aimed at controlling repeated behavior and aggression.

**Figure 7:**
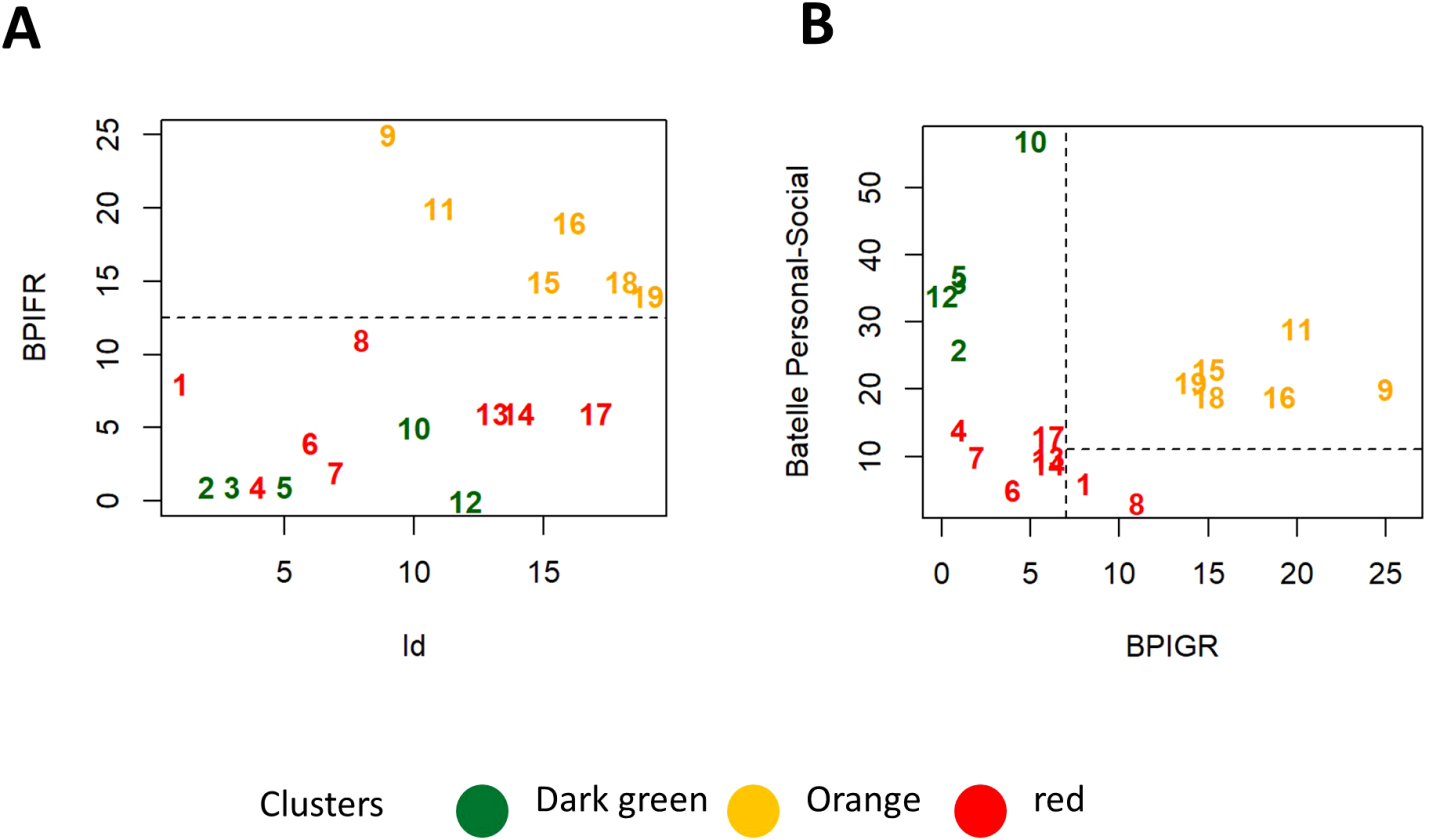
**(A, B)** Scatterplots representing two alternative strategies for identification of patients from the “O” cluster based on BPI-FR alone **(A)** or a combination of Battelle Personal-Social and BPI-GR **(B)**.

We additionally decided to perform tree-based classification, to identify the combinations of scores and sub-scores that more efficiently distinguish patients from the orange (“O”) cluster from the other two sub-groups (“R” and “G”). Using this binary and automated approach, we can see that patients from the “O” cluster can be identified based on a BPI-FR score higher than 12.5 (**Figure 7A**) or a combination of Batelle (Personal-Social subscale) and BPI-GR (respectively Battelle-PS > 11 and BPI-GR > 7, **Figure 7B**). These thresholds are similar to those used for classification according to the “G”,”O”, “R” system, further confirming that patients from the “O” subgroup can be identified based on specific disease features.

### 3.2 “Functional” segregation in the cohort

We previously proposed a numerical “functional” score, called Global Functional Assessment of the patient (GFAP), based on a prioritization array of different “core” clinical weighted- Human Phenotype Ontology (HPO) items (5) to explain the huge clinical and genetic heterogeneity observed in patients with PMS. In fact, our previous data showed a positive correlation between deletion size and GFAP, as well as, that several clinical features mapped preferentially in specific regions after a cluster analysis (by using deletion size; 5).

**Figure 8:**
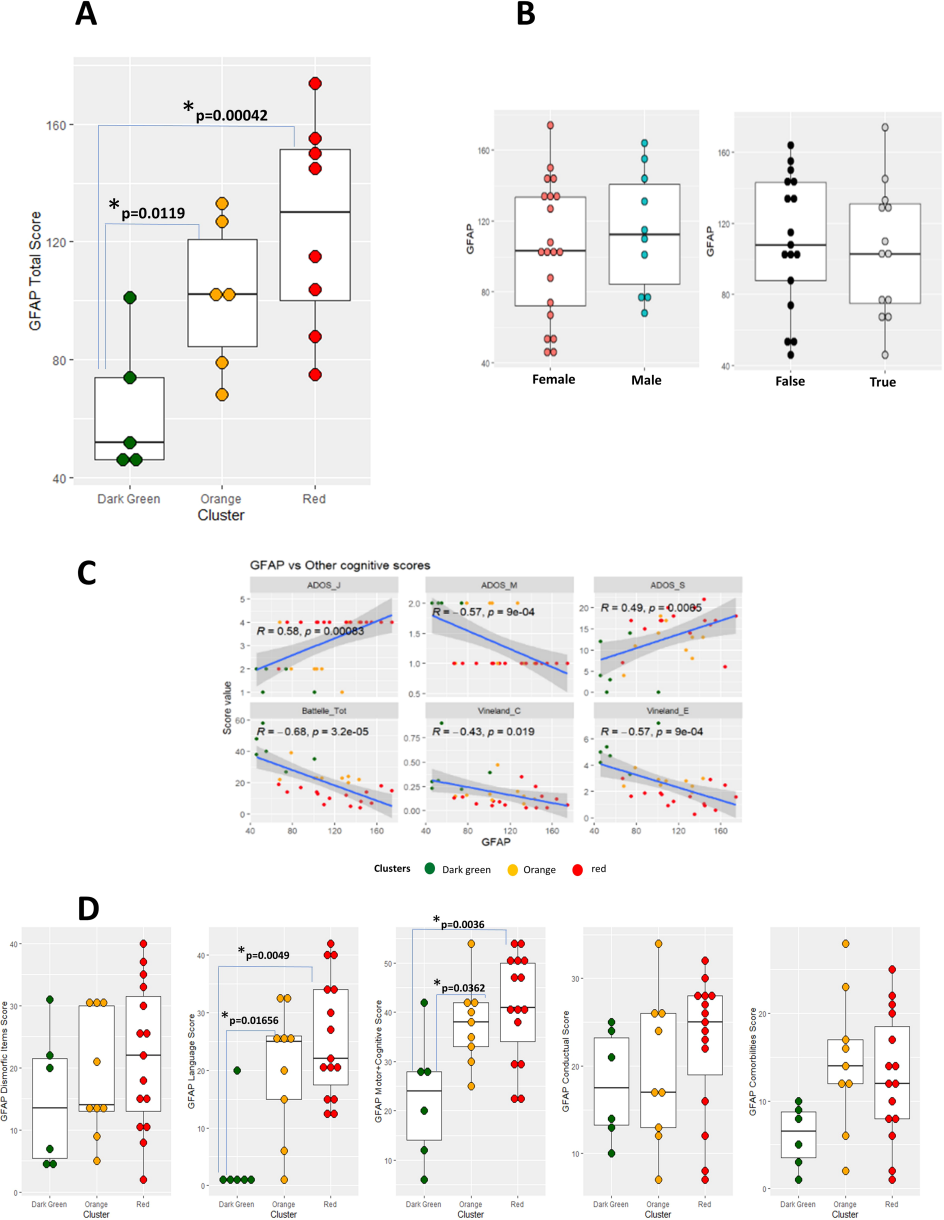
**(A–C)** Box plots representing GFAP total score calculated from patients 1-19. Cluster **(A)**, gender (**B**, left) or sedation status (**B**, right) is reported on the horizontal axis and color coded accordingly. Results of Bonferroni-corrected t test to compare the GFAP values among the three clusters are reported inside **Figure 8A**. **(C)** Panel of scatterplot to identify correlations between GFAP and other cognitive scores (Battelle Mental Age, ADOS-2 and Vineland subscales). **(D)** Box plot representing GFAP subscales from patients 1-19. Coefficient of regression, p-value and regression line with confidence interval are reported in the figure.

We next checked whether GFAP may be used as a Clinical Outcome Assessment (COA) for predicting the cluster segregation observed above, and further characterize the functional differences between the three groups of patients identified. To evaluate its efficacy, the total GFAP scores of the patients were compared by ANOVA analysis and t-test with Bonferroni correction. Statistically significant differences in GFAP scores were detected only comparing the “G” and “O” clusters and the “G” and “R” clusters (p<0.05) (**Figure 8A**). The p-value obtained when comparing the “O” and “R” clusters did not reveal statistical differences (p-value>0.05), although by the visual tendency, the three groups can be separated using the GFAP in combination with total Batelle scale (**Figure 8C**). No differences in GFAP total score were detected comparing patients classified based on gender either sedation status (**Figure 8B**). A correlation with Battelle Mental Age, ADOS (some subscales) and Vineland (some subscales) scores was observed (**Figure 8C**).

**Figure 9:**
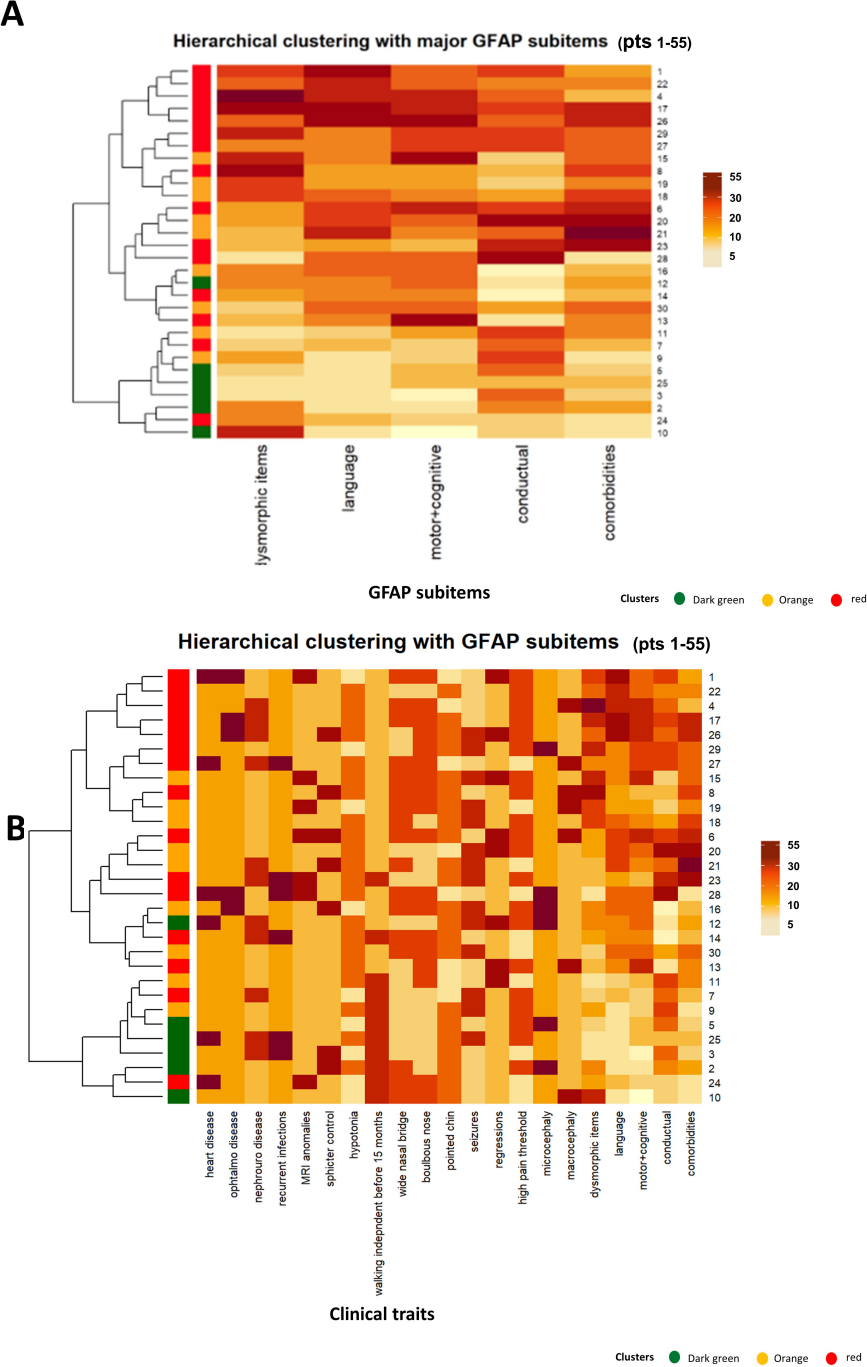
Heatmaps representing GFAP subscale scores **(A)** or individual sub-items **(B)** in the examined PMS patients. Cluster of origin (according to the “ROG” system) is reported on the left, together with a dendrogram illustrating the hierarchical organization of the data.

As GFAP is a multi-construct based on; comorbidity items, developmental delay, speech delay, dysmorphic features, and behavior items, it provides a comprehensive framework for assessing the complex phenotypic variability observed in individuals with the disorder. Thus, we also reevaluated the role of GFAP sub-items to check if any of the latter was in accordance with the groups defined. GFAP sub-items scores of the patients were compared by ANOVA analysis and t-test with Bonferroni correction. None of the sub-scales evaluated showed statistically significant differences between the “R” and “O” clusters, and some of them did not show statistically significant differences between any of the 3 clusters. Indeed, the only sub-scales that make a distinction between the “G” and “R” clusters and between the “G” and “O” clusters are GFAP-Language and GFAP-Motor + Cognitive (Tables below each panel, in **Figure 8D**). If either GFAP subscales scores (**Figure 9A**) or selected sub-items (**Figure 9B**) are considered for unsupervised hierarchical clustering, no clear separation between patients from the three clusters appear. These observations were supported by visualization after PCA dimensionality reduction, considering both the first two and three main components (data not shown).

## 4 Discussion

The highly heterogeneous clinical expression in PMS subjects causes that each affected individual may show unique clinical and behavioral features, presenting challenges in the diagnosis and management of these patients. Therefore, the identification of clinical PMS subgroups is a challenging, but it can be very useful for clinical management and potential benefits from new treatments. Hierarchical unsupervised clustering and PCA were applied to selected psychometric scales to identify subgroups of PMS patients, characterized by specific diseases features. This approach allowed to identify a subset of patients (cluster “O”; orange) characterized by a less severe cognitive impairment but increased aggression or repeated behavior. This specific subgroup of patients could potentially benefit from targeted treatments, such as vafidemstat, aimed at reducing aggression and improving social conduct. The statistical analyses presented here support this hypothesis, however results should be evaluated in the light of the small number of patients in the training and validation sets, common for studies performed on rare or ultra-rare disorders (PSM prevalence is 2-10 of every 1 million of live births). In fact, these findings should be interpreted with caution due to the small sample size and wide age range (5.2–40 years) of participants, which may introduce confounding effects from developmental and aging-related factors. Previous studies, such as Dille et al. (40) or others such Burdeus-Olavarrieta et al. (14) have shown distinct age-related regression or other clinical item patterns, respectively in PMS, underscoring the need for stratified age analyses in future research. Furthermore, the lack of observed effects from age (at evaluation), comorbidities, or medication use may reflect sample heterogeneity (see 5) rather than an absence of such effects in the broader PMS population.

While IQ data were not collected, cognitive functioning was indirectly assessed through validated adaptive and behavioral measures. These tools, such as the Battelle Developmental Inventory and Vineland Scales, provided robust proxies for cognitive abilities and facilitated meaningful group stratification, and they have been previously validated in PMS individuals (14). The clustering algorithm’s reliance on multi-domain inputs minimizes the impact of missing IQ data, although future studies could benefit from additional cognitive metrics. Thus, we stress the importance of developing specific measurement tools to address current limitations, along with ensuring precise psychiatric evaluations to identify potential emotional dysregulations. It is critical to establish effective psychotherapeutic strategies and enhance pharmacological treatments, as well as to understand how this population responds to existing medications. This includes determining whether they might benefit from novel, individualized treatments currently being developed. Such advances would enable more personalized pharmacological and psychotherapeutic care. Additionally, we underline the necessity of longitudinal studies to provide a clearer understanding of developmental trajectories and clinical courses in Phelan-McDermid syndrome, as well as to better anticipate possible comorbidities. Personalized Medicine takes into account the individual characteristics of each patient. It is based on the idea that each person is unique and responds differently to specific treatments, due to their genetic background and other aspects, such as environmental and/or lifestyle factors. Genetic testing is mandatory to identify specific genetic variants, mostly single nucleotide variants (SNVs) that may influence disease susceptibility, clinical features, response to certain medications, and a treatment’s safety or efficacy. Here we show for the very first time in PMS that computed clinical scores can be used to select a group of patients that may benefit from a targeted pharmacological treatment correcting aggressive behavior. Additionally, the heterogeneity of phenotype observed suggests that other targets besides *SHANK3* may be also involved in the PMS phenotype. The behavioral differences observed among patients from the “orange” cluster make them suitable candidates for treatment with LSD1 inhibitors like vafidemstat, which have been proved beneficial for treating aggression and agitation in clinical and preclinical models (23, 24, 26–28). Our efforts to use GFAP as COA showed that GFAP was not entirely useful in distinguishing the different subgroups of PMS patients, with the exception of separating cluster “G” from patients with a more sever phenotype (clusters “O” and “R”). Perhaps increasing the size of the study may lead to a different conclusion regards the usefulness of the GFAP scale in this context.

The natural continuation of this study would be a pilot clinical trial with vafidemstat in this genetically/cognitive-behavioral defined population (orange cluster). Vafidemstat is a lysine-specific demethylase 1 (LSD1) inhibitor currently in Phase II clinical development, demonstrating a strong safety profile and promising therapeutic potential. Preclinical studies in various animal models, along with clinical trials, have shown its effectiveness in improving memory and cognitive function, as well as reducing agitation and aggression (23). These effects have been observed in diverse conditions, including Alzheimer’s disease, Borderline Personality Disorder, Attention-Deficit/Hyperactivity Disorder, and Autism Spectrum Disorder. Given its broad mechanism of action and favorable safety data, vafidemstat represents a potential first-in-class therapeutic approach for addressing the unique challenges faced by patients with Phelan-McDermid Syndrome (PMS), at least for aggressively, and agitation (23, 24, 26–28). Its ability to target key symptoms, including cognitive deficits and behavioral dysregulation, positions it as a promising candidate for improving quality of life in this underserved population. Further studies are warranted to validate its efficacy and establish its role in personalized treatment strategies for PMS.

### 4.1 Future directions

With this proof-of-concept study, conducted as an initial pilot and validated with additional patients, we highlight the need to alert our colleagues to the importance of patient stratification in Phelan-McDermid syndrome. Grouping patients based on their distinct clinical and genetic characteristics may become increasingly relevant and potentially essential in this era of personalized precision medicine for helping to optimize decisions about which group of patients should be included in a subsequent clinical trials or experimental approach in PMS syndrome. To address the inherent limitations of this proof of concept study, a larger-scale, multi-center study is warranted. International patient registries and local or global advocacy organizations, such as the Phelan-McDermid Syndrome Foundation, represent invaluable resources for expanding recruitment and ensuring broader representation of the PMS population.

## Data Availability

The raw data supporting the conclusions of this article will be made available by the authors, without undue reservation.
