# Supplementary material for: Phenotype and psychometric characterization of Phelan-McDermid syndrome patients: pioneering towards personalized medicine
Source: Front Psychiatry. 2025 Mar 4;16:1511962. doi: 10.3389/fpsyt.2025.1511962 (PMC11913864; doi:10.3389/fpsyt.2025.1511962)
Supplement: Supplementary file 1 [file DataSheet1.pdf]

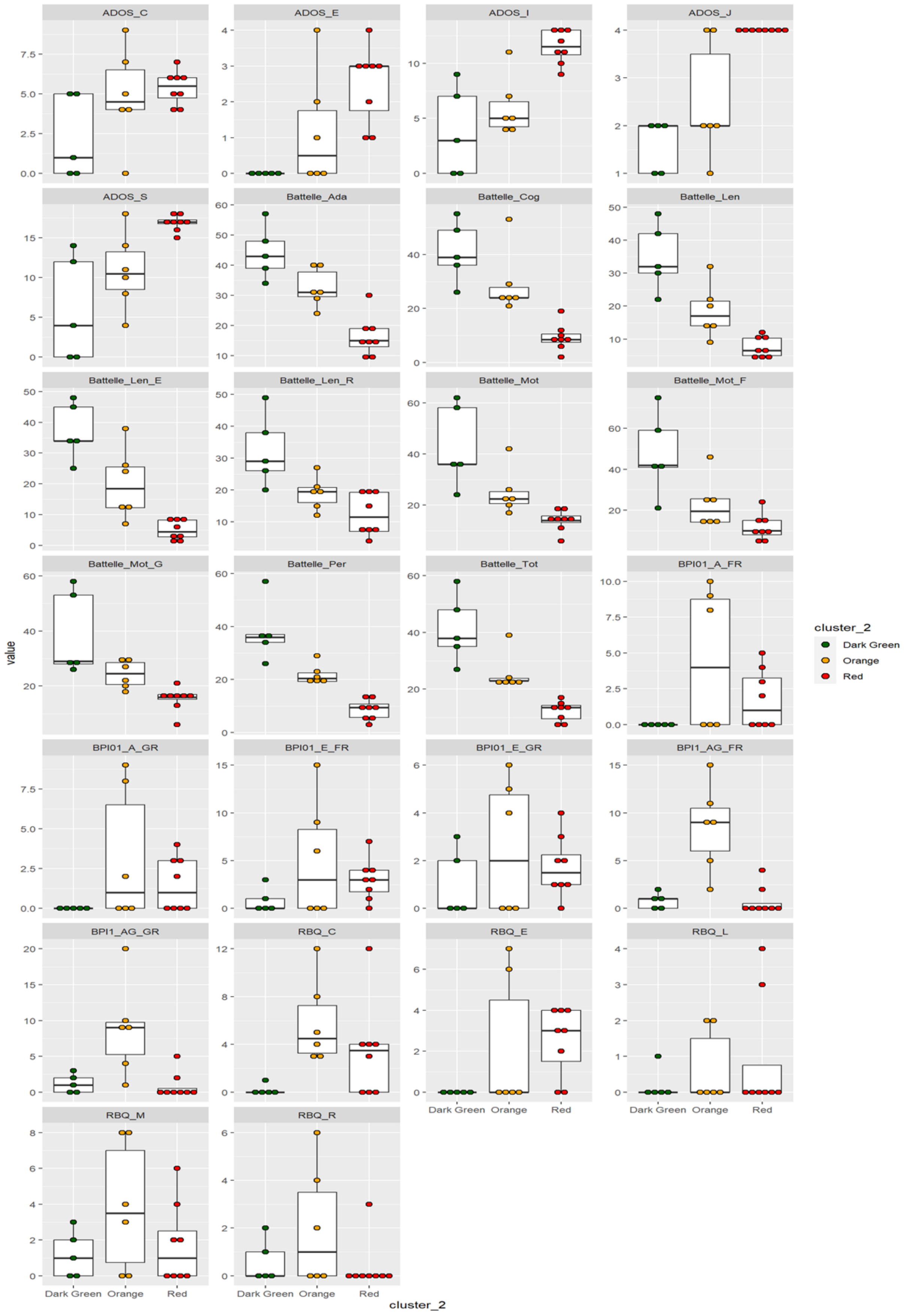

Supplementary Figure 1: Comparison of individual components of psychometric scales between the 3 clusters of patients (color-coded in the figure).
