# Supplementary material for: Phenotype and psychometric characterization of Phelan-McDermid syndrome patients: pioneering towards personalized medicine
Source: Front Psychiatry. 2025 Mar 4;16:1511962. doi: 10.3389/fpsyt.2025.1511962 (PMC11913864; doi:10.3389/fpsyt.2025.1511962)
Supplement: Supplementary file 2 [file DataSheet2.pdf]

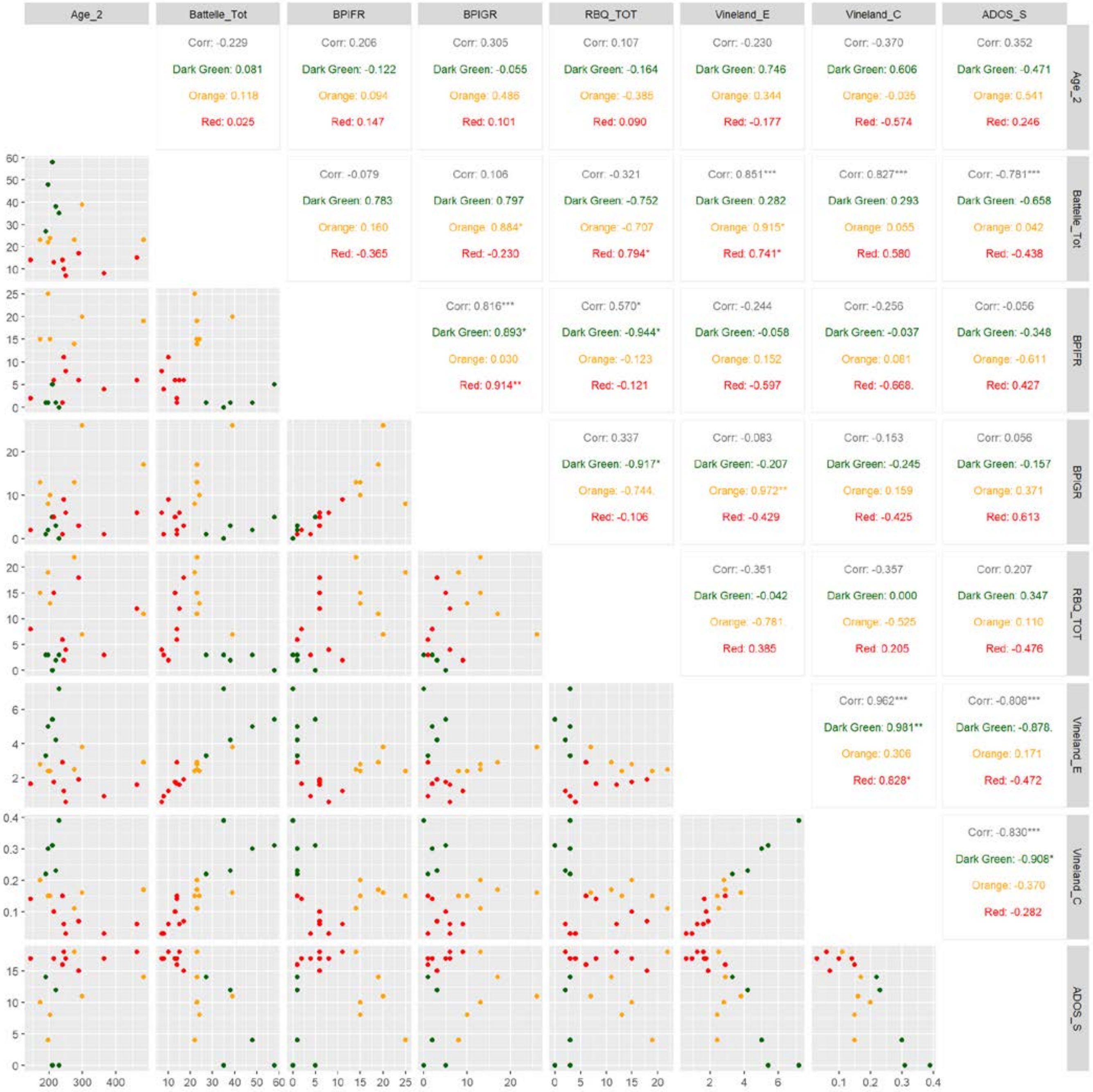

Supplemental Figure 2. A) Scatterplot matrix for different psychometric scales. Person correlation with respective p-values (***: $p < 0.001$, **: $p < 0.01$, *: $p < 0.05$, .: $p < 0.10$) are reported in the upper half of the figure.
